# Overexpression of *ZmWRKY65* transcription factor from maize confers stress resistances in transgenic *Arabidopsis*

**DOI:** 10.1038/s41598-021-83440-5

**Published:** 2021-02-17

**Authors:** Tong Huo, Chang-Tao Wang, Tai-Fei Yu, Da-Ming Wang, Meng Li, Dan Zhao, Xiu-Ting Li, Jin-Dong Fu, Zhao-Shi Xu, Xin-Yuan Song

**Affiliations:** 1 Beijing Advanced Innovation Center for Food Nutrition and Human Health/Beijing Key Lab of Plant Resource Research and Development, Institute of Cosmetic Regulatory Science, Beijing Technology and Business University, Beijing, 100048 China; 2 Institute of Crop Science, Chinese Academy of Agricultural Sciences (CAAS)/National Key Facility for Crop Gene Resources and Genetic Improvement, Key Laboratory of Biology and Genetic Improvement of Triticeae Crops, Ministry of Agriculture, Beijing, 100081 China; 3 Agro-Biotechnology Research Institute, Jilin Academy of Agricultural Sciences, Changchun, 130033 China

**Keywords:** Abiotic, Biotic

## Abstract

Plant-specific WRKY transcription factors play important roles in regulating the expression of defense-responsive genes against pathogen attack. A multiple stress-responsive WRKY gene, *ZmWRKY65*, was identified in maize by screening salicylic acid (SA)-induced de novo transcriptomic sequences. The ZmWRKY65 protein was localized in the nucleus of mesophyll protoplasts. The analysis of the ZmWRKY65 promoter sequence indicated that it contains several stress-related transcriptional regulatory elements. Many environmental factors affecting the transcription of *ZmWRKY65* gene, such as drought, salinity, high temperature and low temperature stress. Moreover, the transcription of *ZmWRKY65* gene was also affected by the induction of defense related plant hormones such as SA and exogenous ABA. The results of seed germination and stomatal aperture assays indicated that transgenic *Arabidopsis* plants exhibit enhanced sensitivity to ABA and high concentrations of SA. Overexpression of *ZmWRKY65* improved tolerance to both pathogen attack and abiotic stress in transgenic *Arabidopsis* plants and activated several stress-related genes such as *RD29A*, *ERD10*, and *STZ* as well as pathogenesis-related (*PR*) genes such as *PR1*, *PR2* and *PR5*; these genes are involved in resistance to abiotic and biotic stresses in *Arabidopsis*. Together, this evidence implies that the *ZmWRKY65* gene is involved in multiple stress signal transduction pathways.

## Introduction

Plants are incapable of long-distance migration and therefore must respond appropriately to ever-changing environmental challenges. Numerous environmental factors, including abiotic and biotic stresses, influence plant development^1,2^. Among them, pathogen attack is one of the most limiting factors that severely threaten crop productivity and quality^3^. The plant innate immune system is composed of two interconnected branches. The first branch involves pathogen-associated molecular pattern (PAMP)-triggered immunity (PTI), which is recognized by plant receptors via a mitogen-activated protein kinase (MAPK) signaling cascade^4,5^. The second branch involves effector-triggered immunity (ETI); ETI is an accelerated defense response and is recognized by plant resistance (R) proteins^5^. R gene-activated ETI involves a complex defense program, including the production of both reactive oxygen species (ROS) and salicylic acid (SA), rapid programmed cell death (hypersensitive responses (HRs)), and the induction of many host genes, including pathogenesis-related (PR) genes^5^. PTI- and ETI-mediated defense responses in plants are modulated mainly by three signaling hormone molecules: SA, jasmonic acid (JA), and ethylene (ET)^6^. Both synergistic and antagonistic interactions occur between SA and JA/ET signaling pathways during the progression of plant immune responses^1,7^. This apparent discrepancy reflects the complexity of plant defense mechanisms^8^.

The transcriptional regulation of defense-related gene expression is central to the induction of disease resistance in higher plants^9^. Several families of transcription factors involved in the transcriptional regulation of plant defense genes have been identified, including those consisting of ET-responsive factors (ERFs), Myb-like proteins, bZIP proteins, or WRKY proteins^1,2,10^. Among these families, the WRKY family is one of the largest transcription regulatory families among plants. WRKY proteins are characterized by their conserved DNA-binding WRKY domains, which consist of a highly conserved WRKYGQK stretch at the N-terminus and a zinc-finger motif (C-X_4-5_-C-X_22-23_-H-X_1_-H or C-X_7_-C-X_23_-H-X_1_-C) at the C-terminus^11,12^. WRKY transcription factors are divided into three groups based on the number of WRKY domains and zinc-finger motifs^3,11^. Group I proteins have two WRKY domains, each containing a C_2_H_2_ zinc-finger motif; group II proteins embody one WRKY domain and a C_2_H_2_ motif; and group III proteins contain a single WRKY domain and a C_2_H_2_ motif^11,13^.

Pathogen treatments can lead to the selective up-regulation of several WRKYs^14–17^. WRKYs bind specifically to W-box sequences containing an invariant TGACC/T core sequence; these W-boxes are in the promoters of many plant defense-related genes^18–20^. Accordingly, WRKYs are involved in defense responses. 49 WRKY genes in *Arabidopsis* are induced by SA or pathogens^21^. And *Magnaporthe grisea* induced 15 WRKY genes in rice^22^. At least 13 *Oryza sativa* WRKY (OsWRKY) genes are known to positively regulate rice resistance against pathogens such as *M. oryzae*, *Rhizoctonia solani* and *Xanthomonas oryzae* pv. *oryzae* (Xoo)^3,23–26^. The transcriptional regulatory mechanisms of WRKYs during the induction of the defense response have been well studied^16,27^. Plant MAPK cascades function via WRKYs to regulate downstream gene expression during the defense response^28^. By releasing *AtWRKY33* in the nucleus, *AtMPK4* regulates the expression of defense genes such as *PAD3*^29^. The AtWRKY33 protein is also phosphorylated by AtMPK3/AtMPK6^30^. OsWRKY53 functions downstream of the wound-responsive OsMKK4-OsMPK1 cascade to improve tolerance to rice blast fungus^31^. In *Nicotiana benthamiana, WRKY8* plays a role in defense response and is phosphorylated by SIPK, NTF4, and WIPK^32^.

Maize (*Zea mays* L.) is widely cultivated in temperate and tropical zone of the world. In these regions, the environmental conditions are diminishing, especially due to pathogen attack, which severely affect grains development and productivity. WRKY genes mediate abiotic and biotic stress responses in plants, but little information about the mechanisms of WRKYs in maize^33^. *ZmWRKY106* and *ZmWRKY40* were found to improve the tolerance to drought and high-temperature stress^34,35^. In the present study, we identified a multiple stress-responsive WRKY gene, *ZmWRKY65*, by screening a SA-induced de novo transcriptomic sequence of maize. Overexpression of *ZmWRKY65* in transgenic *Arabidopsis* plants improved tolerance to both pathogen attack and drought.

## Results

### Drought- and SA-induced de novo transcriptomic sequence analyses of maize

**Figure 1:**
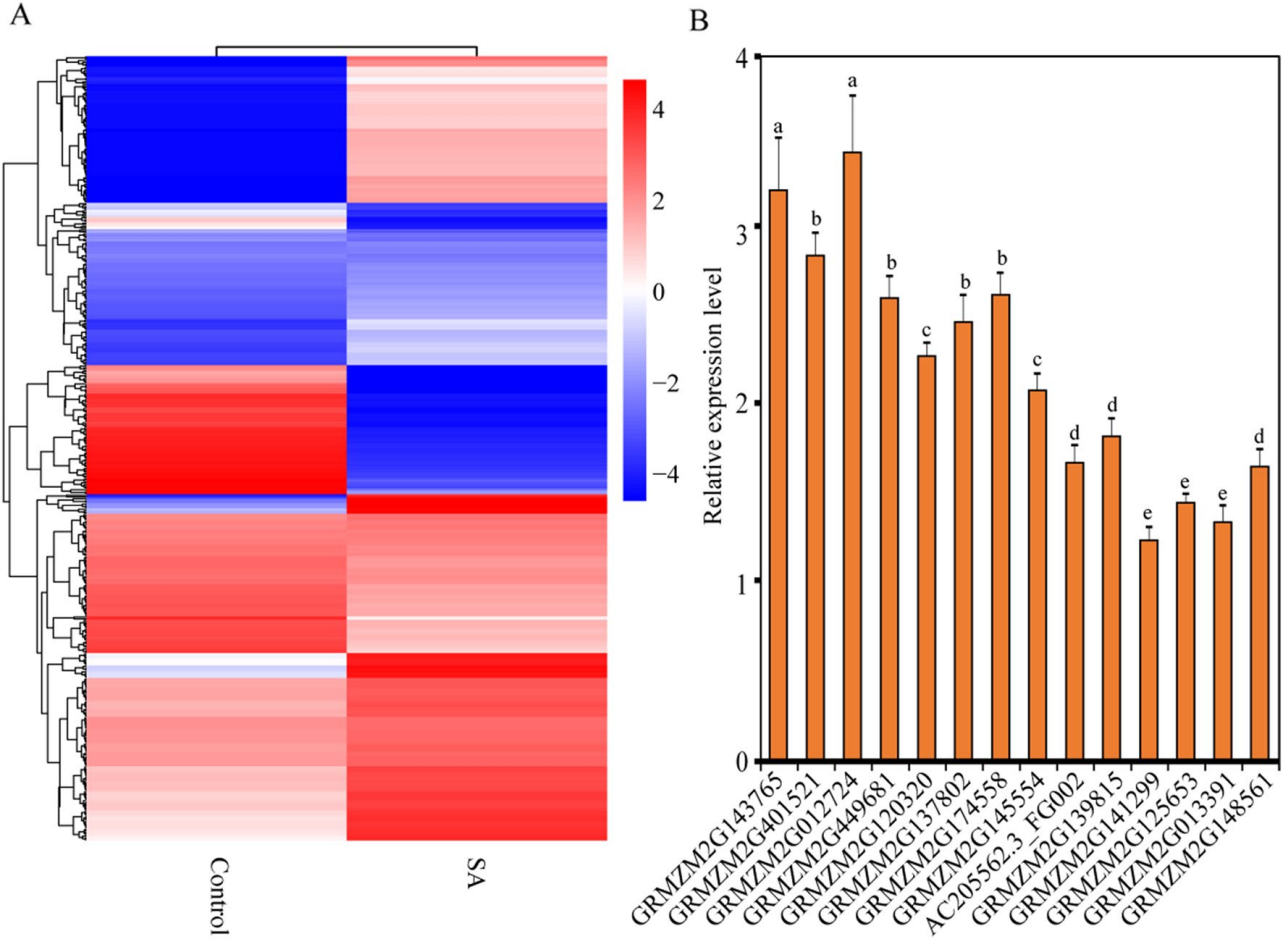
De novo transcriptomic sequence analyses of maize under SA stresses. (**A**) The cluster analyses of differential expression genes under SA treatments. (**B**) the transcription levels of the 14 WRKY transcription factors under SA treatments. Vertical bars indicate ± SE of three replicates.

De novo transcriptomic sequence analyses (De novo transcriptome sequencing of maize SRP144573, https://www.ncbi.nlm.nih.gov/search/all/?term=SRP144573)34 of maize showed, the transcription levels of many genes had been changed under stress treatment (Fig. 1A). Transcription factors became the prime candidates to study, played important roles in regulating the expression of stress-related genes in plants. The results showed a large number of transcription factor genes existed differential expressions before or after the treatment. WRKY transcription factors were reported to response to biotic and abiotic stresses among these transcription factors^36^. Analysis found that 14 WRKY transcription factors were induced to up-regulated by drought^34^ and SA treatments (Fig. 1B). The 14 ZmWRKY gene family members in Fig. 1B were renamed in order from GRMZM2G143765 to GRMZM2G148561^35^. Among these 14 WRKY transcription factor, *ZmWRKY65*, had the highest expressions than others under drought and SA treatments and was chosen for further study. The result of the KEGG pathway database showed the ‘signal transduction of plant hormone’ and ‘interaction between plant and pathogen’ pathway enriched the most DEGs under drought and SA treatments, respectively (Figure S1A and S1C). The result of Gene Ontology classification showed the 60 predominant GO classifications were presented in Figure S1B and S1D. Signaling and response to biotic stimulus in Figure S1B and S1D were enriched and among the predominantly enriched groups.

### Phylogenetic analysis and domain organization of the putative maize WRKY65

**Figure 2:**
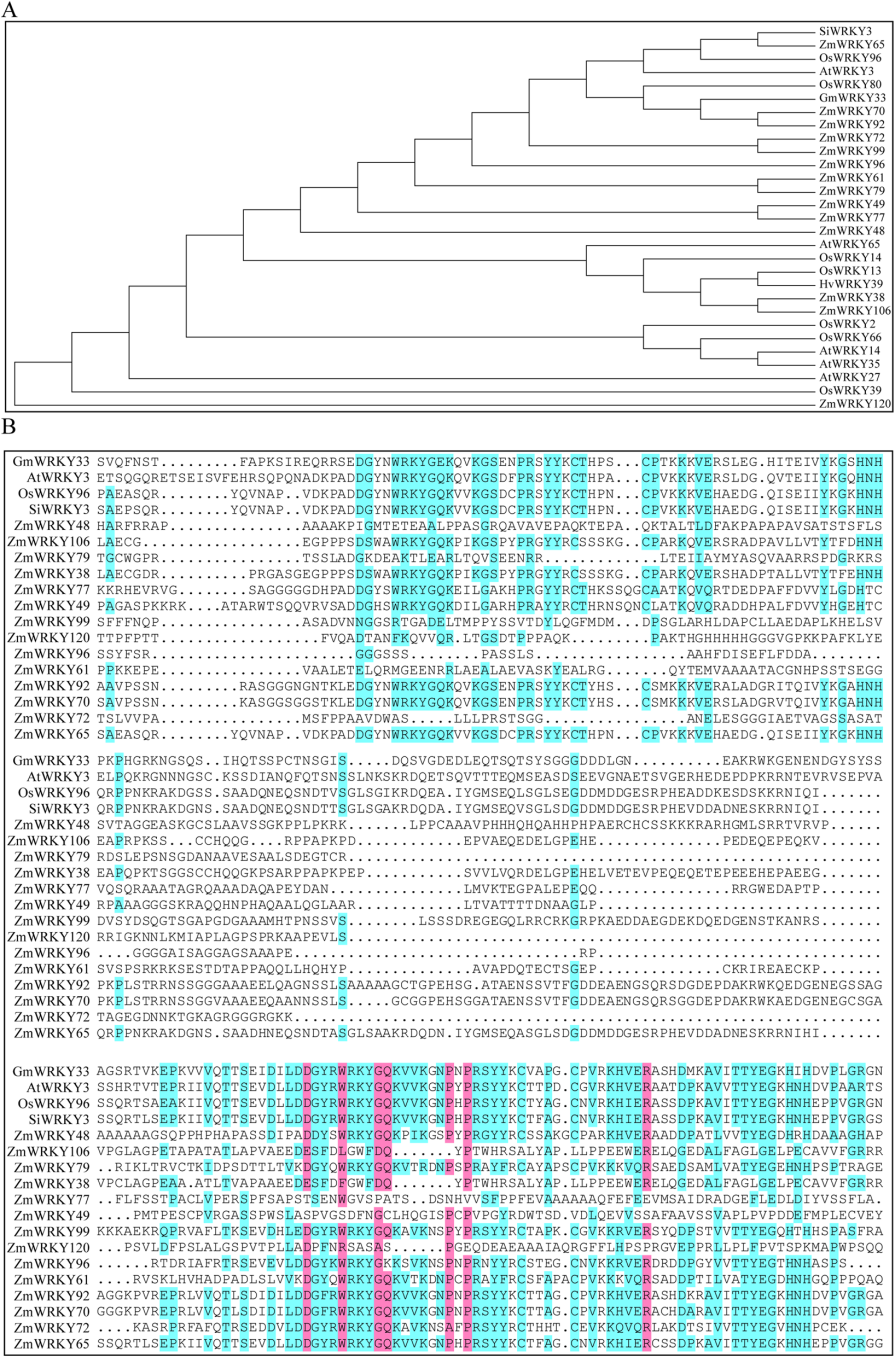
Analyses of sequence alignments and phylogenetic tree. (**A**) Phylogenetic analysis of ZmWRKY65 in relation to closely related WRKY proteins. (**B**) Alignment of the putative amino acid sequence of ZmWRKY65 with sequences from *Arabidopsis*, rice, millet, barley and soybean.

*ZmWRKY65*, a putative WRKY member selected from the drought- and SA-treated maize de novo transcriptomic data, contains a 1548-bp open reading frame (ORF) that encoded 516 amino acids and contains a predicted WRKYGQK domain coupled with an N-terminal coiled-coil domain (amino acids 102–142) as well as a zinc-finger motif (C–X_5_–C–X_23_–H–X_1_–H). WRKY protein sequences of fifteen available high homology from various species and the 14 maize WRKY transcription factors from drought- and SA-induced de novo transcriptomic sequence were downloaded from the Phytozome 12.1 public database (https://phytozome.jgi.doe.gov/pz/portal.html), and the program MEGA 5.1 was used to establish a neighbor-joining phylogenetic tree (Fig. 2A). The highly homologous sequences with ZmWRKY65 from the phylogenetic tree and 14 maize WRKY transcription factor sequences were selected for further analysis, and the results of multiple amino acid sequence alignments also showed that *Arabidopsis* AtWRKY3, rice OsWRKY96, millet SiWRKY3 and soybean WRKY33 shared the highest genetic similarity with ZmWRKY65, indicating possible functional similarity among them (Fig. 2B).

### Analysis of the *ZmWRKY65* promoter

To gain insight into the mechanism responsible for the transcriptional regulation of *ZmWRKY65*, we isolated a 2.0-kb promoter region upstream of the *ZmWRKY65* ATG start codon. According to the Plant *Cis*-acting Elements database (http://www.dna.affrc.go.jp/PLACE/), several stress response-related *cis*-acting elements are present in the promoter region (Table S1). Several regulatory elements responding to drought, abscisic acid (ABA), cold and salt were also recognized, including ABA-responsive element (ABRE PyACGTGG/TC) MYB-responsive element (MYB TGGTTAG) MYC-responsive element (MYC CACATG) dehydration- responsive element (DRE CCGAC) low temperature- responsive element (LTRE CCGAC) recognition site sequences (Figure S2). Overall, the results of this analysis suggest that *ZmWRKY65* probably functions in the abiotic stress response.

### Subcellular localization and expression pattern analyses of ZmWRKY65

**Figure 3:**
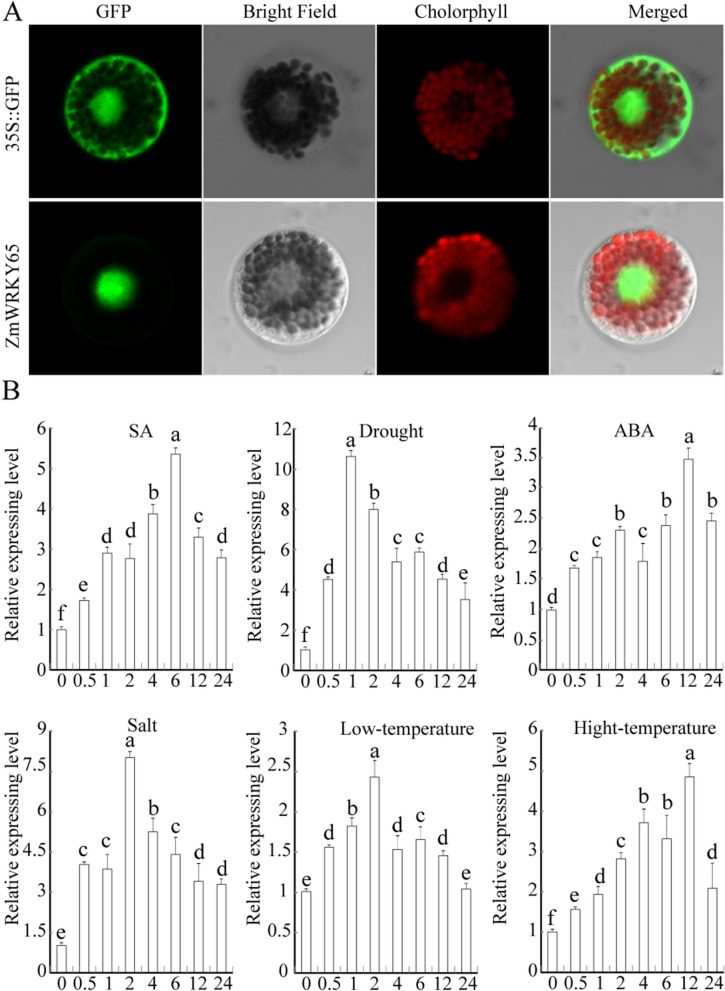
Molecular characteristic analysis of *ZmWRKY65* gene. (**A**) Green fluorescent signals of control (GFP) and ZmWRKY65-GFP were detected in wheat epidermal cells with a laser confocal scanning microscope. (**B**) The expression patterns of the *ZmWRKY65* gene under different stress treatments. *ZmWRKY65* up-regulation was induced by hormones (ABA and SA) and responded to drought, salt, high-temperature and low-temperature stresses. The results shown are averages of three replicates. Vertical bars indicate ± SE of three replicates.

To determine the subcellular localization of ZmWRKY65, using the PEG-mediated method, we transformed ZmWRKY65-GFP constructs under the control of the CaMV 35S promoter into wheat mesophyll protoplasts. The subcellular localization of green fluorescent protein (GFP) expression in the wheat mesophyll protoplasts was subsequently observed. At the same time, the hGFP reporter construct was transformed into wheat mesophyll protoplasts, which served as controls. Relative to the hGFP reporter construct, the ZmWRKY65::hGFP fusion protein was localized in the nucleus (Fig. 3A). The nucleus, cytoplasm and cell membrane proteins of wheat mesophyll protoplasts were extracted for western blot analysis. The result showed the ZmWRKY65-GFP signal were detected in nucleus, however, the control GFP were detected in nucleus, cytoplasm and cell membrane (Figure S3).

To further investigate the response of *ZmWRKY65* to abiotic stresses, we performed quantitative real-time PCR (qRT-PCR) using RNA isolated from stress-treated maize plants. The results showed that *ZmWRKY65* could be induced by drought, NaCl, high temperature, low temperature, SA and ABA (Fig. 3B). *ZmWRKY65* rapidly responded to drought and peaked (more than tenfold) at 1 h after treatment. Under salt stress, the transcription level of *ZmWRKY65* increased gradually, peaked at a level 8.87-fold greater than that of the control at 2 h and then rapidly decreased to a level similar to that of the control. Under high-temperature stress, *ZmWRKY65* transcript levels were up-regulated and peaked at 4.98-fold greater levels at 12 h. In response to exogenous ABA treatment, *ZmWRKY65* transcript levels were up-regulated and peaked at 3.38-fold greater levels at 12 h.

### Effects of ABA and SA on the germination of transgenic *Arabidopsis*

**Figure 4:**
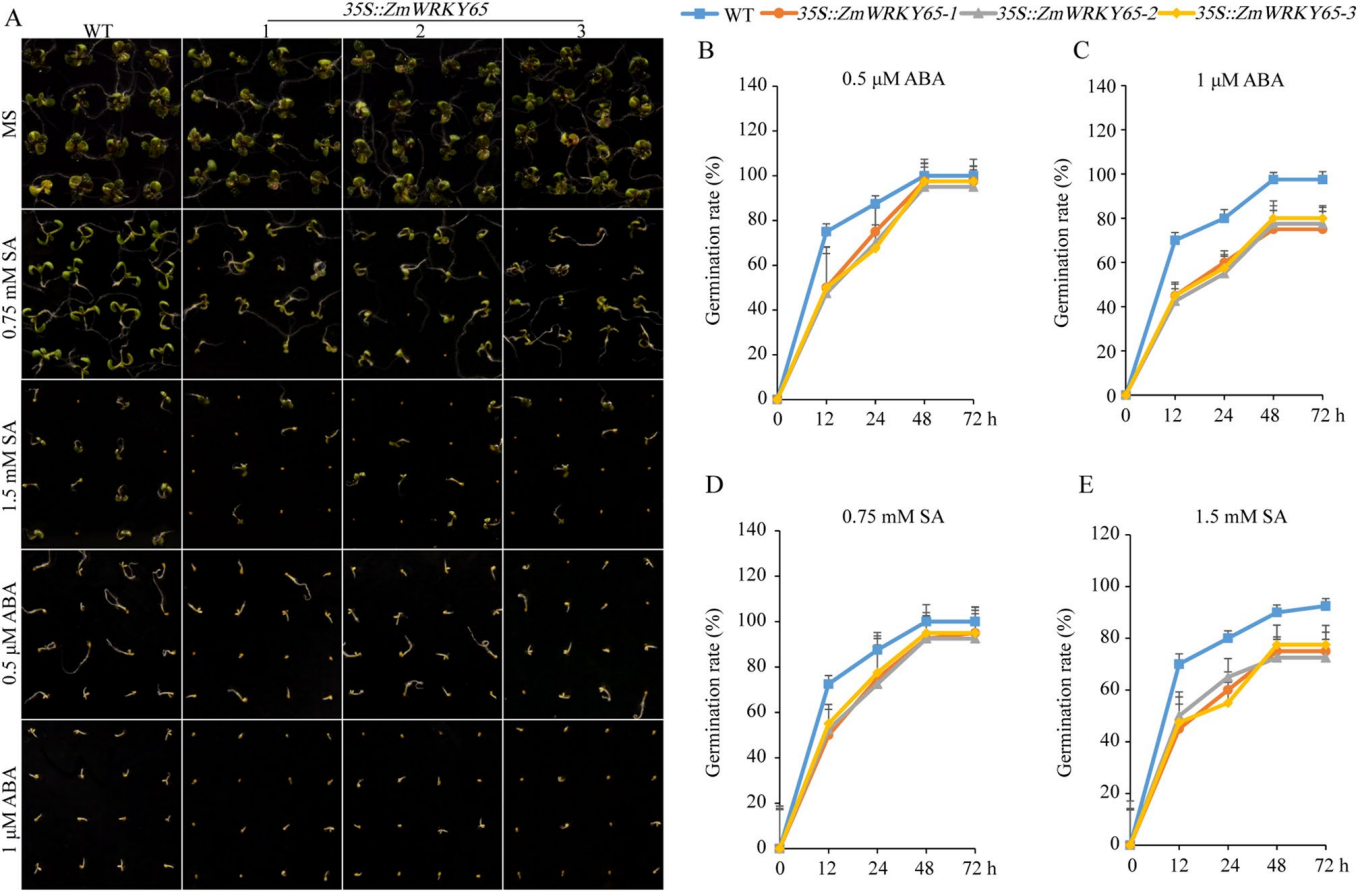
Germination of transgenic *Arabidopsis* plants and WT under different hormone treatments. (**A**) The germination phenotypes of WT and *ZmWRKY65* transgenic *Arabidopsis* plants under different hormone treatments. (**B**) The germination rates of WT and *ZmWRKY65* transgenic *Arabidopsis* plants under 0.5 μM ABA treatment. (**C**) The germination rates of WT and *ZmWRKY65* transgenic *Arabidopsis* plants under 1 μM ABA treatment. (**D**) The germination rates of WT and transgenic *ZmWRKY65 Arabidopsis* plants under 0.75 mM SA treatment. (**E**) The germination rates of WT and *ZmWRKY65* transgenic *Arabidopsis* plants under 1.5 mM SA treatment. The results are averages of three replicates. Vertical bars indicate ± SE of three replicates.

To determine the sensitivity of transgenic *ZmWRKY65 Arabidopsis* plants to ABA and SA, germination assays were carried out. Three T3 homozygous lines that exhibit relatively high transcriptional levels of *ZmWRKY65* in transgenic *Arabidopsis* were selected for further analysis. The wild-type (WT) and transgenic *Arabidopsis* line seeds were placed on 1/2MS medium supplemented with various concentrations of ABA and SA. The results showed that the transgenic *ZmWRKY65 Arabidopsis* lines were more sensitive than WT lines were to ABA and high concentrations of SA (Fig. 4A). In response to treatment with exogenous ABA and high concentrations of SA, the growth of WT and transgenic *Arabidopsis* lines was inhibited, and the transgenic *Arabidopsis* lines had lower germination rates than did the WT lines (Fig. 4B, 4C, 4D and 4E). Furthermore, at 72 h after high concentrations of ABA and SA treatment, approximately 70% of the transgenic *Arabidopsis* seeds germinated. At that point, however, approximately 90% of the WT seeds germinated (Fig. 4).

### *ZmWRKY65* improves stress tolerance in transgenic *Arabidopsis*

**Figure 5:**
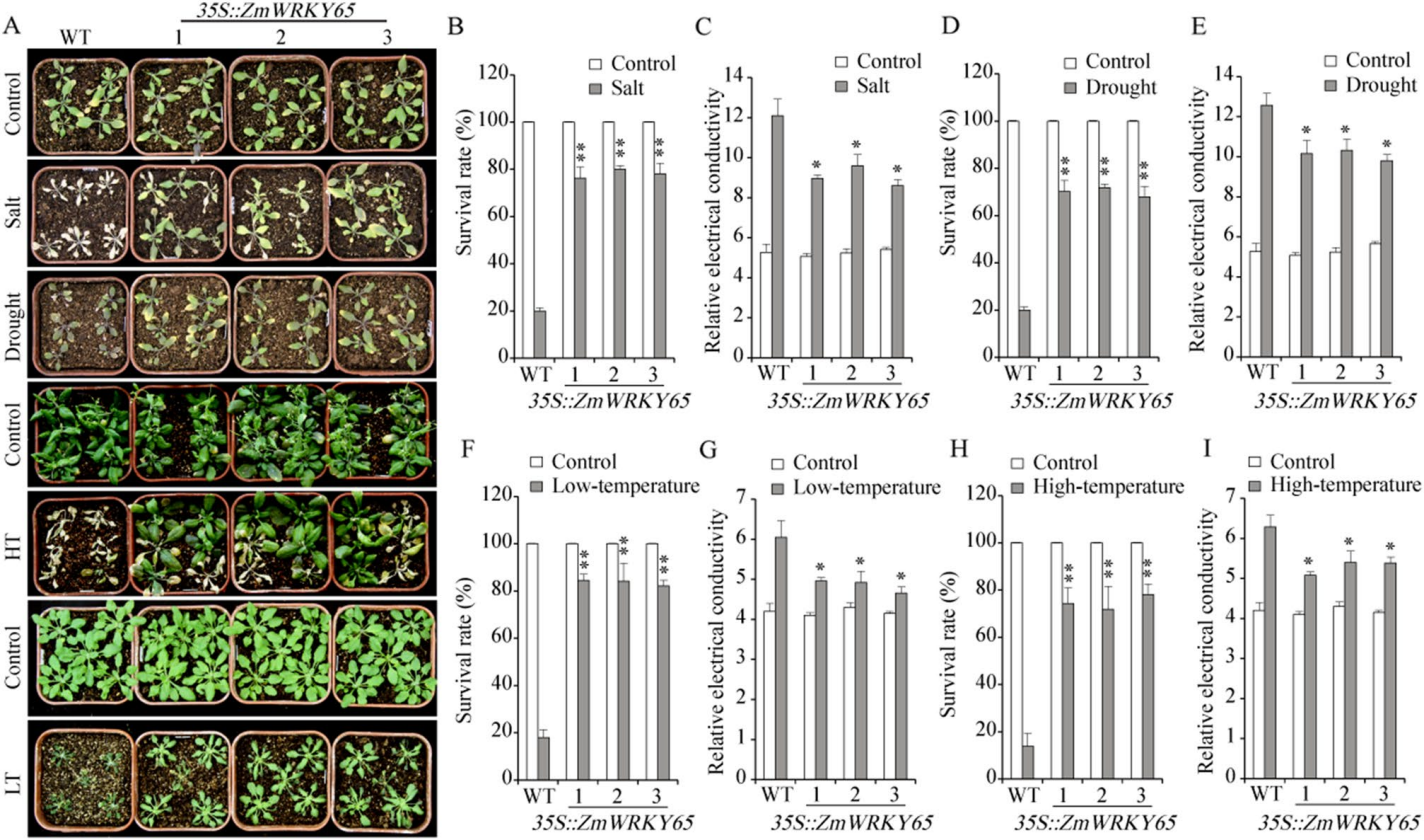
Stress tolerance of WT and transgenic *Arabidopsis* plants under different treatment conditions. (**A**) The phenotypes of WT and transgenic *ZmWRKY65 Arabidopsis* plants under different stress treatments. (**B**) and (**C**) The survival rates and relative electrical conductivities of WT and *ZmWRKY65* transgenic *Arabidopsis* plants under salt treatments. (**D**) and (**E**) The survival rates and relative electrical conductivities of WT and *ZmWRKY65* transgenic *Arabidopsis* plants under drought treatments. (**F**) and (**G**) The survival rates and relative electrical conductivities of WT and *ZmWRKY65* transgenic *Arabidopsis* plants under low-temperature treatments. (**H**) and (**I**) The survival rates and relative electrical conductivities of WT and *ZmWRKY65* transgenic *Arabidopsis* plants under high-temperature treatments. The results are averages of three replicates. Vertical bars indicate ± SE of three replicates. *, ** indicate significant differences in comparison with the WT lines at 0.01 < P < 0.05 and P < 0.01, respectively.

To verify the tolerance of transgenic *Arabidopsis* plants to abiotic stresses, drought, salt, low temperature and high temperature were imposed on three-week-old T3 homozygous lines. Under non-stressed conditions, no differences were observed in survival rate or relative electrical conductivity between the transgenic and WT plants (Fig. 5A). However, after the stress treatments, the significant differences were observed between the transgenic and WT plants: the transgenic *Arabidopsis* plants had a higher survival rate (Fig. 5B, D, F, H) and a lower relative electrical conductivity (Fig. 5C, E, G, I) than did WT plants. Under the stress treatments, approximately 60% of transgenic *Arabidopsis* plants survived, and only approximately 15% of WT plants survived.

### Effects of ABA on stomatal movement and the water loss rate

**Figure 6:**
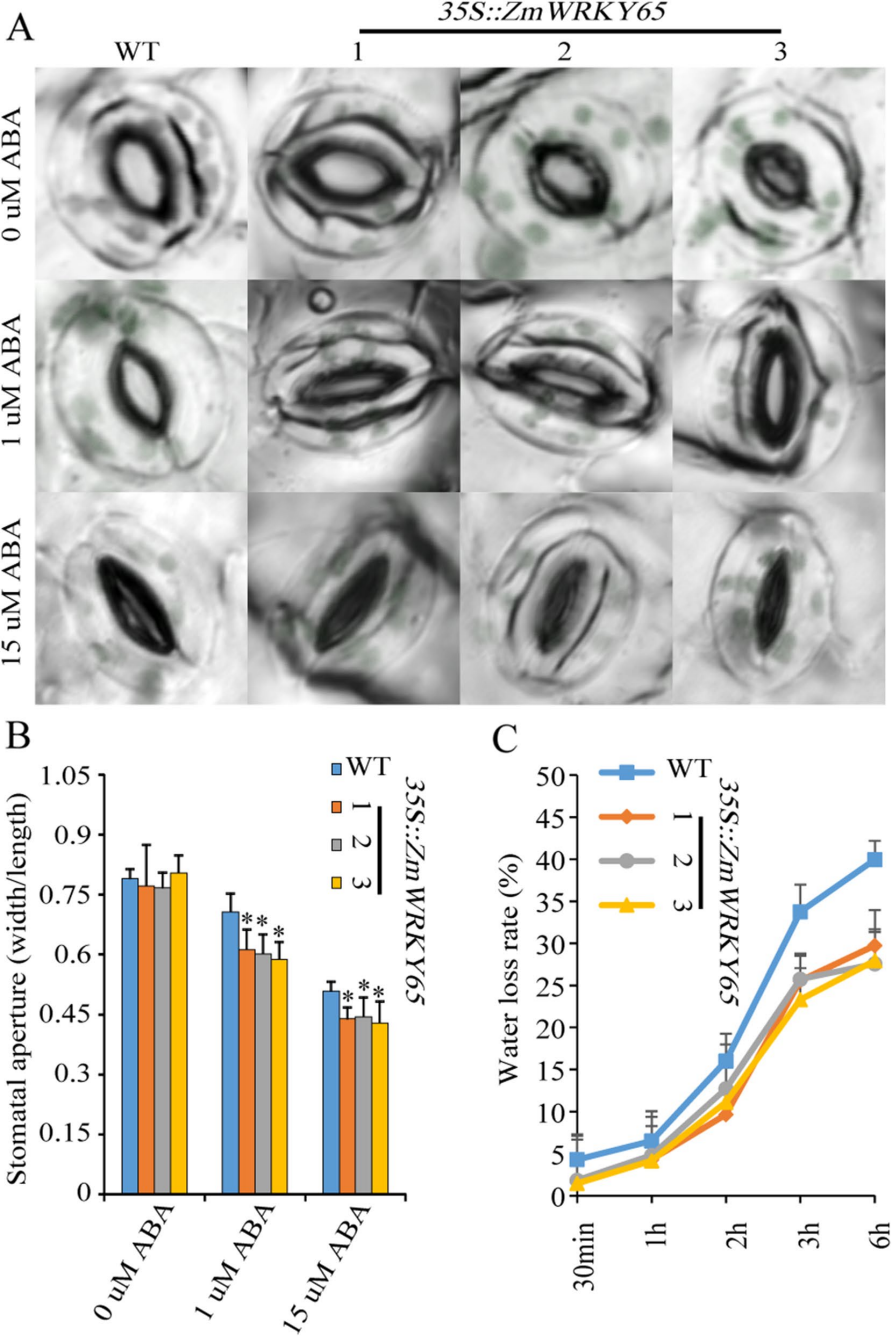
Effects of ABA on stomatal aperture and water loss rate. (**A**) The phenotypes of WT and *ZmWRKY65* transgenic *Arabidopsis* plants under different concentrations of ABA treatment. (**B**) The stomatal aperture of WT and *ZmWRKY65* transgenic *Arabidopsis* plants under different concentrations of ABA treatment. Vertical bars indicate ± SE of three replicates. * indicates significant differences in comparison with the WT lines at 0.01 < P < 0.05. (**C**) Water loss rate. Vertical bars indicate ± SE of three replicates.

To further analyze the effects of ABA on transgenic *Arabidopsis* plants, stomatal aperture sizes were assayed. As shown in Fig. 6, the mean stomatal apertures of the control and transgenic *Arabidopsis* leaves decreased concurrently as the concentrations of ABA increased, and following treatment with exogenous ABA, the stomata of the transgenic *Arabidopsis* leaves closed faster than did the stomata of the control leaves. This result provides more evidence that overexpression of *ZmWRKY65* in *Arabidopsis* improves sensitivity to ABA. This result provides more evidence that overexpression of *ZmWRKY65* in *Arabidopsis* improves sensitivity to ABA. The transgenic *Arabidopsis* leaves showed lower rates of water loss during dehydration than did the control leaves (Fig. 6C). For example, at the 0.5 h time point of dehydration, the control *Arabidopsis* leaves revealed a water loss of 4.3%, whereas the transgenic *Arabidopsis* leaves revealed a loss of 1.6% (average of the three transgenic lines); such differences in water loss between the WT and transgenic plants persisted and grew larger as time progressed. At the 6-h time point of dehydration, the control *Arabidopsis* leaves revealed a water loss of 39.9%, whereas the transgenic *Arabidopsis* leaves revealed a loss of 28.4% (average of the three transgenic lines).

### Overexpression of *ZmWRKY65* improves resistance against *Botrytis cinerea* and *Pst*. DC3000 in transgenic plants

**Figure 7:**
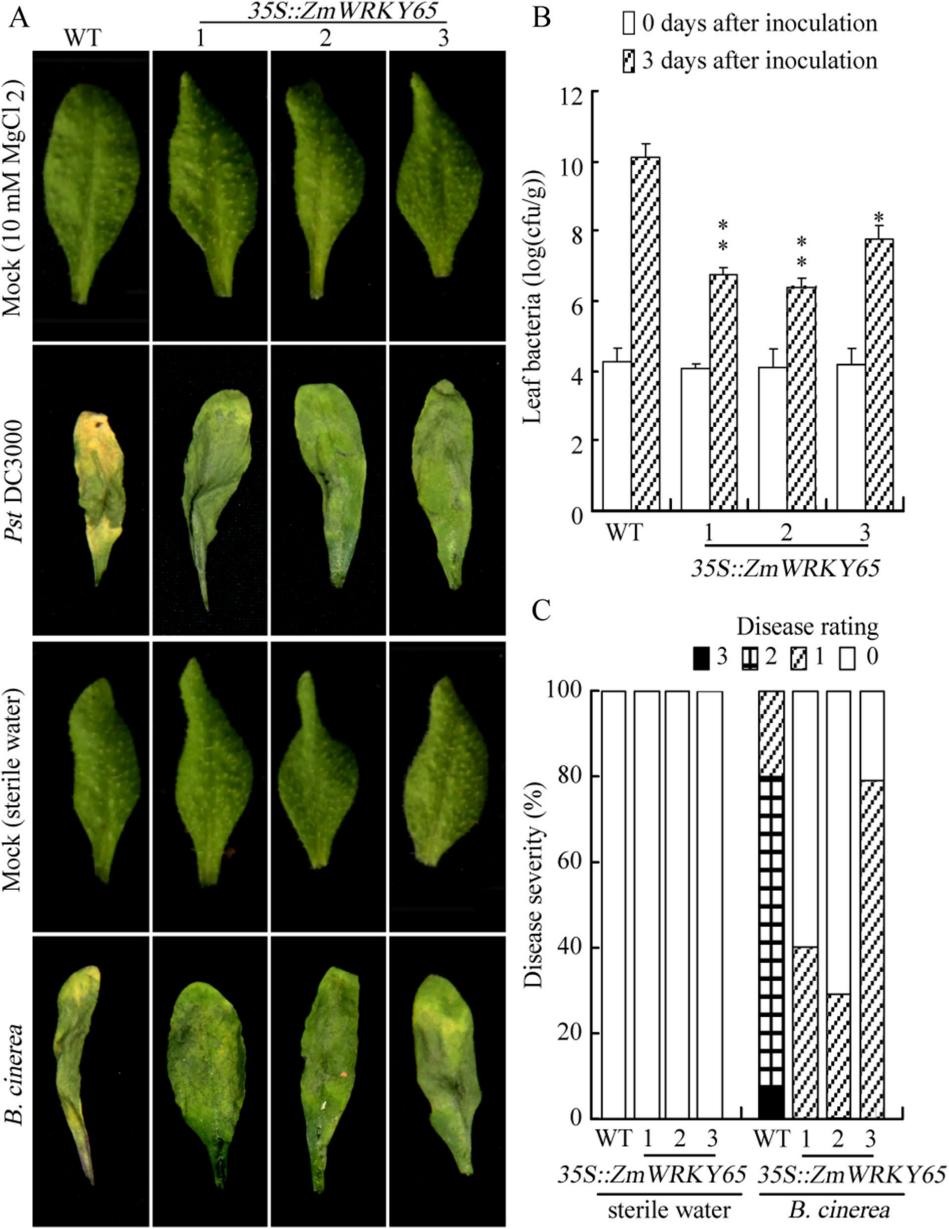
Fungal and bacterial pathogen tolerance of transgenic plants. (**A**) The phenotypes of WT and *ZmWRKY65* transgenic *Arabidopsis* plants under bacterial and fungal treatments. (**B**) Bacterial pathogen (*P. syringae*) response of three independent transgenic *Arabidopsis* lines and WT. The bacterial population was investigated 3 days after the plants were inoculated with 107 CFU/mL of *Pst*. DC3000. The results are averages of three replicates. Vertical bars indicate ± SE of three replicates. *, ** indicate significant differences in comparison with the WT lines at 0.01 < P < 0.05 and P < 0.01, respectively. (**C**) Histogram of fungal disease (*B. cinerea*) symptoms of three independent transgenic *Arabidopsis* and WT lines 8 days after the plants were inoculated with sterile water or with 2 × 10^5^ conidiospores/mL. 0–3 represent the disease rating: 0, no infection/necrosis; 1, 1–4 leaves showing some necrosis; 2, 5–10 leaves showing necrosis; 3, dead plants.

To analyze the disease resistance of transgenic *Arabidopsis* plants, 3-week-old seedlings of transgenic and WT *Arabidopsis* plants were investigated. As shown in Fig. 7A, overexpression of *ZmWRKY65* in *Arabidopsis* improved disease resistance, and when *Arabidopsis* leaves (pretreated with water) were inoculated with *Pst.* DC3000 and *B. cinerea*, severe disease symptoms and extended hyphae were observed 3 days after inoculation with *Pst.* DC3000 and 8 days after inoculation with *B. cinerea*. After being inoculated with *Pst.* DC3000, the leaves of WT plants were wilted and dried up on the third day; however, the leaves of transgenic plants showed mild wilting (Fig. 7A). A similar phenotype was observed 8 days after inoculation with *B. cinerea*. The results of the statistical analysis showed that at 3 days post-infection, the bacterial titres of the three transgenic lines were significantly lower than those of the WT lines (Fig. 7B). Similarly, the results of the disease severity tests showed that 57% of the transgenic *Arabidopsis* plants (average of the three transgenic lines) exhibited first-degree disease symptoms at 8 days after inoculation with *B. cinerea*; however, 71% of WT plants exhibited second-degree disease symptoms, and 9% exhibited third-degree disease symptoms (Fig. 7C). Meanwhile, the changes of SA level were monitored in transgenic *Arabidopsis* and WT plants. The results showed no obvious difference was observed between the WT and transgenic *Arabidopsis* plants under the MgCl_2_ treatment, but the inoculation of isolated leaves with pathogens suspension resulted in an increase in the SA level, and significantly higher SA levels were detected in the three transgenic lines compared with WT plants (Figure S4).

### Overexpression of *ZmWRKY65* affected the transcription of various PR- and stress-responsive genes

**Figure 8:**
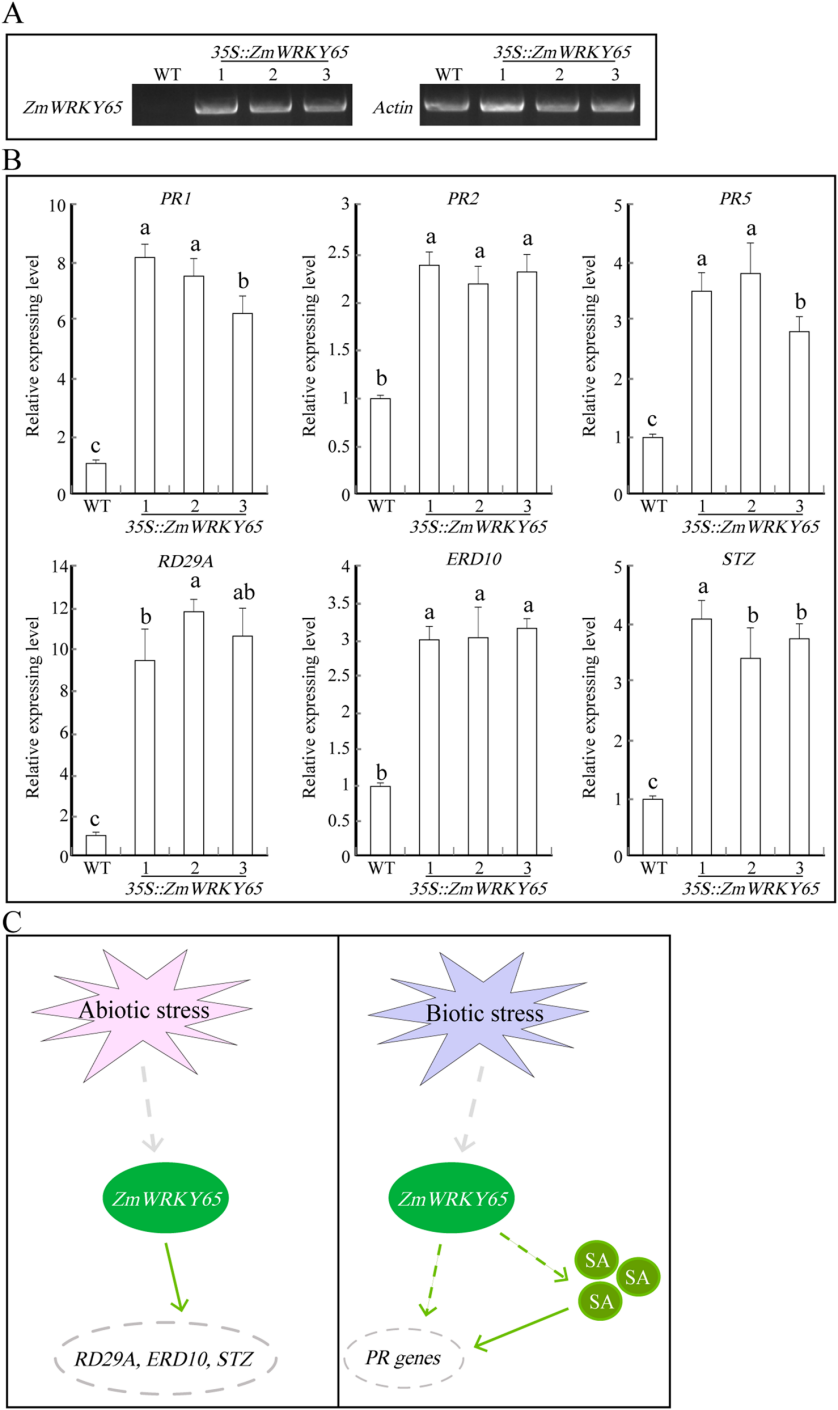
Expression analysis of stress-responsive genes in *ZmWRKY65* transgenic *Arabidopsis*. (**A**) The transcription levels of *ZmWRKY65* in transgenic *Arabidopsis* plants. (**B**) The transcription levels of stress-related genes in transgenic *Arabidopsis* plants. *Arabidopsis Actin* (ACT2) was used as an internal control. Vertical bars indicate ± SE of three replicates. (**C**) The mode of *ZmWRKY65* gene regulation.

In order to analyze molecular mechanisms of *ZmWRKY65* in different stress responses, the expression patterns of stress-related genes in transgenic *Arabidopsis* and WT lines were detected under normal conditions. As shown in Fig. 8, T3 homozygous transgenic *ZmWRKY65 Arabidopsis* plants were tested by semi-quantitative PCR, and the result showed that *ZmWRKY65* could overexpress in transgenic *Arabidopsis* (three amplified clear and repeating bands) (Fig. 8A). Meanwhile the transcripts of disease resistance PR genes such as *PR1*, *PR2*, and *PR5* genes were found to up-regulated in transgenic lines (Fig. 8B). In addition, overexpression of *ZmWRKY65* also increased the transcription of stress related genes such as *ERD10*, *STZ* and *RD29A* (Fig. 8B). These results revealed that *ZmWRKY65* activated the expressions of disease- and stress-responsive genes in transgenic *Arabidopsis* plants.

## Discussion

Transcriptional regulation exert effect on the process of plant-inducible defense responses activation. Therefore, regulatory components identification and transcriptional regulatory pathways of the plant defense system establishment were high on the agenda. Thus far, multiple transcription factor families about plant defense-related genes have been identified, including ERF, Myb-like, bZIP, and WRKY families. Compared with other transcription factor families, the WRKY family has a much greater proportion of genes related to biotic stress responses, suggesting that the WRKY family fulfill a valuable function in responding to biological stress^27^. Most knowledge about the functions of WRKY genes in defense-related signal transduction pathways come from the analysis of dicot plants, such as *Arabidopsis*^21^, tomato^37^, tobacco^38^ and potato^39^; very limited information has been reported in maize^40^ or other monocots. It is therefore important to elucidate of the functions of WRKY proteins involved in maize defense response signaling.

Emerging evidence has highlighted the importance of WRKY transcription factors acting as positive or negative regulators in plant disease resistance networks^41^. Global gene expression profiling has revealed a large number of WRKY genes rapidly induced or repressed upon pathogen infection, suggesting that WRKY genes may contribute to the regulation of responses to pathogen infection in rice^22,42,43^. Subsequent data analysis established that overexpression of *ZmWRKY65*, which was screened from the SA-induced de novo transcriptomic sequencing data of maize, could slow down the rate of wilting to enhance disease tolerance in *Arabidopsis* and increase the expression of disease-responsive genes (PR genes). In addition, many defense or defense-related genes, including well-studied PR genes and the regulatory *NPR1* gene, contain in their promoters W-box elements that are specifically recognized by WRKY proteins and are necessary to induce the expression of those genes^44–46^. It has recently been reported that, by directly binding to the promoter of the defense-related gene *OsPR10a*, OsWRKY51 functions as a positive transcriptional regulator in defense signaling against Xoo^25^. Overexpression of *PtrWRKY89* in transgenic poplar plants, which were more susceptible to both hemibiotrophic and necrotrophic pathogens, results in constitutive expression of PR genes^47^. *AtWRKY70* was identified as a common regulatory component in SA- and JA-dependent defense-signaling pathways and mediates the cross-talk between these antagonistic pathways downstream of *NPR1*^48^. These results implied that WRKY transcription factors played an important role in disease resistance.

SA-mediated signaling pathways are important for disease resistance. The accumulation of SA can induce local defenses to stop pathogenic invasion and activate systemic acquired resistance^7,49^. Despite many studies reporting that SA-regulated gene transcript levels increase in *Arabidopsis* following the onset of disease, the use of SA biosynthetic and signaling mutants have not clearly revealed the roles of SA in *Arabidopsis* defense^50^. Experiments on transgenic plants have demonstrated various roles for SA in pathogen defense, such as basal defense to the fungal pathogen *B. cinerea*^51^. Our results showed that overexpression of *ZmWRKY65* resulted in enhanced plant resistance to *B. cinerea* and *Pst.* DC3000 and improved expressions of SA-regulated PR genes, including *PR1*, *PR2* and *PR5* (Fig. 8B), these genes have been reported in enhancing the disease-resistant capacity of plants^24,52–55^. These results suggested that overexpression of *ZmWRKY65* could promote the synthesis of SA, which resulted in the rapid accumulation of SA (Fig. 8C and Figure S4) and improved disease tolerance.

Many studies have revealed that WRKY genes are activated and up-regulated by drought, salt and exogenous hormones in a time-dependent manner, indicating that WRKY transcription factors are involved in the response to abiotic stress. In maize, the genes *ZmWRKY40* and *ZmWRKY106* were identified to enhance the tolerances to drought and high-temperature^34,35^. The protein structure analysis showed that there was the conservative amino acid sequence between *ZmWRKY65* and *ZmWRKY106* (Fig. 2B)*,* and this result implied *ZmWRKY65* may had the similar function in stress resistance with *ZmWRKY106.* To verify the roles of *ZmWRKY65* in response to abiotic stress, transgenic *Arabidopsis* plants were exposed to drought, salt, high temperature and low temperature. Overexpression of *ZmWRKY65* enhanced stress tolerance of the transgenic plants and the expression patterns of some stress-responsive genes such as *STZ*^56,57^, *ERD10*^58^ and *RD29A*^59^ (Fig. 8B, C). The accumulation of the plant hormone ABA is induced by drought, which contributes to stomatal closure and therefore prevents water loss^60^. Our results showed that transgenic plants displayed greater sensitivity to ABA than did WT plants when the leaves of both types of plants were treated with ABA; the ABA treatment resulted in rapid stomatal closure, possibly reducing water loss rates (Fig. 6) and improving drought tolerance. Hence, the *ZmWRKY65* gene is likely an important component for abiotic and biotic signal transduction pathways and encodes a multifunctional factor that can integrate different stress signals in plants.

## Materials and methods

### De novo transcriptome sequence analysis of maize

Four-leaf stage maize inbred line B73 (From Chinese Academy of Agricultural Sciences, Beijing) seedlings grown under normal conditions (25 °C ± 2 °C temperature, 60–70% relative humidity, 18 h light/6 h dark photoperiod, and natural sunlight) were washed off with water, and then the seedlings dehydrated on filter paper for 4 h and 5 mM SA for 4 h treatment, then the samples (each sample had the three repetitions) were collected for transcriptome sequence analysis^61^. Vertical bars indicate ± SE of three replicates. The detailed process of transcriptome sequence analysis was exhibited as previous description.

### Plant materials and stress treatments

Seeds of maize inbred line B73 were germinated in an incubator and then transferred to pots (10-cm diameter, 10 seedlings per pot) containing a vermiculite: soil mixture (1:1, v/v). The seedlings were grown under the following conditions: 25 °C ± 2 °C temperature, 60–70% relative humidity, 18-h light/6-h dark photoperiod, and natural sunlight. For drought treatments, three-leaf-stage maize seedlings were dried and dehydrated on filter paper at 25 °C and 20% relative humidity. For NaCl treatments, the seedling roots were immersed in 250 mM NaCl. For hormone treatments, the leaves of maize seedlings were sprayed with different hormones (100 μM ABA and 5 mM SA). After being treated, the seedlings were harvested at different time points, and the specific methods were described by Wang et al. (2018)^34^.

### RNA extraction and qRT-PCR

The maize tissue total RNAs were isolated by using the method was described by Yu et al.^61^, and first-strand cDNAs were synthesized using a PrimeScript First Strand cDNA Synthesis kit (TaKaRa, Japan). The cDNAs were combined with SYBR master mix (TIANGEN, China), and an ABI7300 system (Applied Biosystems, USA) was used to monitor the kinetics of the PCR products for qRT-PCR (consist of 94 °C for 3minutes, and then 40 cycles of 94 °C for 30 s, 60 °C for 15 s, 72 °C for 34 s). The maize *actin* gene (GRMZM2G126190) was used as an internal control for normalization of the template cDNA. The amount of accumulated transcript of the *ZmWRKY65* gene normalized to the internal *actin* control gene was determined using the 2^−ΔΔCT^ method. The qRT-PCR primers of *ZmWRKY65* were provided in Table S2. Each sample PCR was repeated three to four biological replicates.

### Gene isolation and sequence analysis

The *ZmWRKY65* opening reading frames were obtained from the maize cDNA. The PCR products were cloned into pEASY-T1 vectors (TransGen) and then sequenced by using an ABI 3730XL 96-capillary DNA analyzer (Lifetech). Sequence alignment was performed by ClustalX using BioEdit software, and the sequences were adjusted manually. The neighbor-joining method was used to construct a phylogenetic tree by the MEGA5.1 program, and the confidence level of monophyletic groups was estimated using a bootstrap analysis of 1000 replicates^62^.

### Subcellular localization in protoplasts

For the subcellular localization analysis, the expression vector p16318GFP with a GFP tag was used. The methods about *ZmWRKY65-*hGFP recombinant vector construction were described by Yu et al.^61^, after which then transient expression vector *ZmWRKY65-*hGFP and the control vector p16318GFP were transformed into common wheat mesophyll protoplasts in accordance with the PEG-mediated method^63^. After being cultured for 16 h in darkness, the wheat protoplasts were monitored by confocal microscopy to determine the subcellular localization of GFP expression. Specifically, GFP fluorescent signals were observed with a confocal laser scanning microscope (Nikon); FM4-64 dye (Molecular Probes, Carlsbad, CA) was excited at 543 nm, and its fluorescence was recorded using a 650-nm long-pass filter.

### Generation of transgenic *Arabidopsis*

The fragment of *ZmWRKY65* was amplified and cloned into a modified pBI121 vector under control of the CaMV 35S promoter, resulting in a *35S::ZmWRKY65* construct. *Arabidopsis thaliana* ecotype Col-0 plants were grown under normal growth conditions (16 h light/8 h dark photoperiod, 23 °C temperature, 60–70% relative humidity) until flowering. And we used the *Agrobacturium*-mediated transformation method to generate the transgenic *Arabidopsis* lines^61^. The three T3 transgenic lines were chosen by semi-quantitative PCR for further analysis. The detection result was placed in Fig. 8A.

### Performance of transgenic *Arabidopsis* under stress treatments

For the different hormone treatments, one hundred homozygous T3 transgenic lines and control plants seeds (which were subjected to 3 days of vernalization treatment) were placed on 1/2MS solid medium that were supplemented with different hormone treatments (0.5 μM ABA, 1 μM ABA, 0.75 mM SA and 1.5 mM SA) for 4 days under normal conditions (16 h light/8 h dark photoperiod, 23 °C temperature, 70% relative humidity); the germination rates were recorded during 4 days of treatment. Transgenic T3-generation *Arabidopsis* lines were used to perform stress tolerance assays. Three-week-old *Arabidopsis* plants grown in soil (including those subjected to 3 days of vernalization treatment) under normal conditions were stopped watering to expose to drought conditions for one week. After 7 days of drought treatment, the seedling plants were then re-watered and grow in the normal conditions for 7 days of recovery, and then the survival rates were recorded. For salt tolerance assays, 3-week-old *Arabidopsis* plants grown in soil under normal conditions were exposed to salt treatment (irrigation with 200 mM NaCl); after 7 days of salt treatment, the survival rates were recorded. The experiments were repeated three times, and the results were consistent. One set of experiments is shown. For heat and cold tolerance assays, three-week-old *Arabidopsis* plants grown in soil under normal conditions were exposed to high temperature (42 °C) for 12 h and low temperature (-8 °C) for 3 days, and then the survival rates were recorded.

### Tomato (Pst) DC3000 (virulent) fungal infection of transgenic plants

To explore transgenic plant disease resistance, four-week-old *Arabidopsis* plants were treated with *B. cinerea* conidiospores and *Pseudomonas syringae* pv. *tabaci*, respectively. The mock inoculations were carried out with sterile water and 10 mM MgCl_2_, respectively, and the details were described by Xu et al.^17^, Thilmony et al.^65^ and Lu et al.^66^.

### The detection of SA content

Four-week-old seedlings of transgenic *Arabidopsis* and WT plants were treated with pathogens for 3 days, and then 0.5 g leaves with pathogens treatment and 10 mM MgCl_2_ treatment were harvested and ground to fine powder in liquid nitrogen, and then the samples was used for ELISA analysis. The methods of the ELISA assay were described by Lu et al.^66^.

### Relative electrical conductivity

Sample leaves (0.1 g, 3-week-old *Arabidopsis* seedlings) of the transgenic and control lines (under natural growth conditions and subjected to stress treatments) were put into 10 mL of distilled water. To measure the initial electric conductance (S1) by FG3-B FiveGoTM conductivity meter (METTLER TOLEDO, Switzerland) (25 °C), a vacuum (test tube) was applied for 30 min, after which the vacuum pressure surged for 2 h. The applied temperature was then increased (100 °C) for 30 min, after which it was reduced to room temperature (25 °C), at which point the final electric conductance (S2) was determined. The relative electrical conductivity was evaluated as S1 × 100/S2.

### Analysis of stomatal aperture and water loss

Leaves of three-week-old *Arabidopsis* seedlings were collected and incubated for 4 h in stomatal-opening solution (0.05 M KNO_3_, 10 mM 2-(N-morpholino) ethanesulfonic acid (MES), 50 μM CaCl_2_, pH = 6.15) under high-light conditions (810 μmol/m^2^/s^1^, 25 °C), after which the leaves were transferred to an ABA-containing solution (0, 1 μM, and 15 μM) for 2 h. The leaves were then mounted on slides and observed with a confocal laser scanning microscope (CLSM). To measure water loss, leaves of 3-week-old *Arabidopsis* plants were excised and placed on a bench (25 °C, 30% relative humidity); the initial fresh weights of the leaves were measured at 0 h and recorded as W0, and then after 0.5 h, 2 h, 3 h and 5 h, the fresh weights of leaves were measured and recorded as Wn (n = 0.5, 2, 3, and 5). The water loss rate was evaluated as: (W0 − Wn) × 100/W0.

## Supplementary Information

Supplementary Information.

## Data Availability

All the data supporting the findings is contained within the manuscript.
