# Supplementary material for: Overexpression of ZmWRKY65 transcription factor from maize confers stress resistances in transgenic Arabidopsis
Source: Sci Rep. 2021 Feb 17;11:4024. doi: 10.1038/s41598-021-83440-5 (PMC7889854; doi:10.1038/s41598-021-83440-5)
Supplement: Supplementary file 1 — Supplementary Information. [file 41598_2021_83440_MOESM1_ESM.pdf]

Overexpression of *ZmWRKY65* transcription factor from maize confers stress resistances in transgenic *Arabidopsis*

Running title: *ZmWRKY65* gene confers abiotic and disease tolerances in transgenic plants

Tong Huo[1†], Chang-Tao Wang[1†], Tai-Fei Yu[2†], Da-Ming Wang[3], Meng Li[1], Dan Zhao [1], Xiu-Ting Li[1], Jin-Dong Fu[2*], Zhao-Shi Xu[2*], Xin-Yuan Song[3*]

[1]Beijing Advanced Innovation Center for Food Nutrition and Human Health/Beijing Key Lab of Plant Resource Research and Development, Beijing Technology and Business University, Beijing 100048, China

[2]Institute of Crop Science, Chinese Academy of Agricultural Sciences (CAAS)/National Key Facility for Crop Gene Resources and Genetic Improvement, Key Laboratory of Biology and Genetic Improvement of Triticeae Crops, Ministry of Agriculture, Beijing 100081, China

[3]Agro-biotechnology Research Institute, Jilin Academy of Agricultural Sciences, Changchun 130033, China

[†]Authors contributed equally to the present work.

[*]Corresponding author: E-mail: songxinyuan1980@163.com (X-Y S), xuzhaoshi@caas.cn (Z-S X) or fujindong@caas.cn (J-D F)

Figure S1

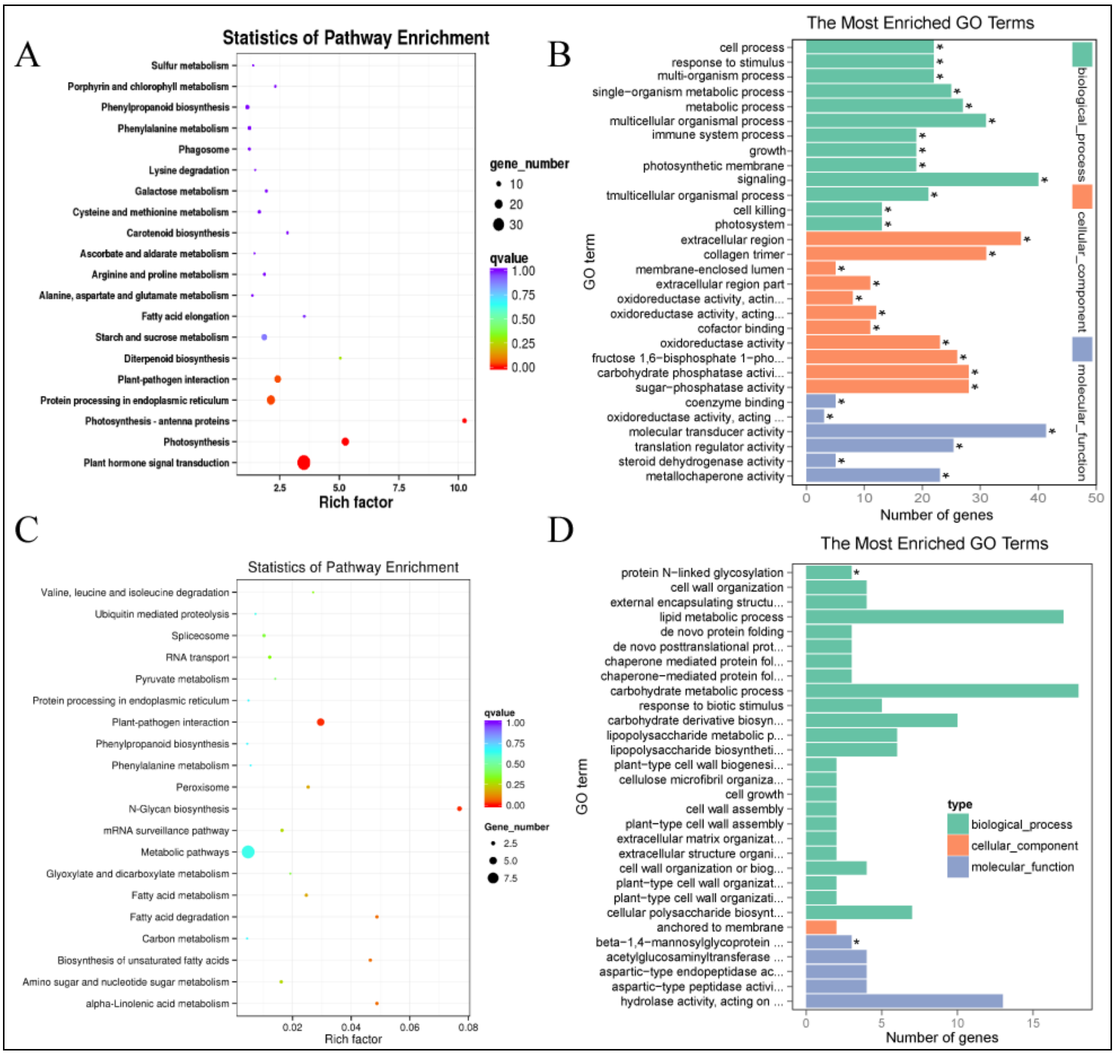

Figure S1 Analysis of the Functional Annotation and Classification the DEGs. (A) and (C) Genes and Genomes enrichments of the annotated DEGs between the control and stressed perennial ryegrass. The left Y-axis indicates the KEGG pathway. The X-axis indicates the Rich factor. A high q-value is represented by blue, and a low q-value is represented by red. (B) and (D) Gene ontology classifications of DEGs between the control and stressed perennial ryegrass. The Y-axis represents the number of DEGs in a category. The results of stressed vs. control are summarized in three main categories: BP, CC, and MF.

Figure S2

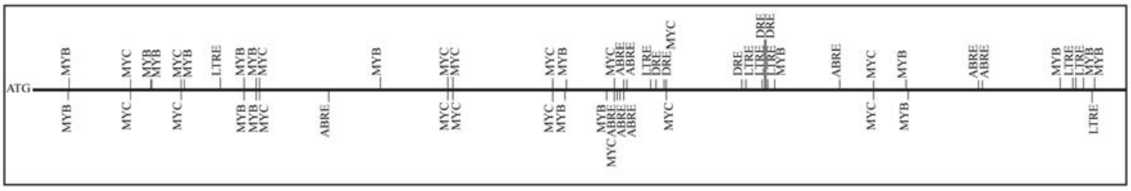

Figure S2 The cis-acting element of the *ZmWRKY65* promoter

Figure S3

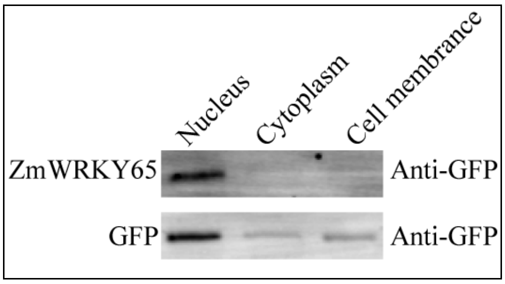

Figure S3 The subcellular location analysis of the ZmWRKY65 by western blot.

Figure S4

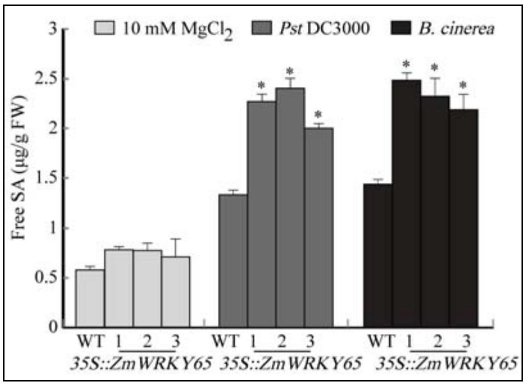

Figure S4 The free SA levels in Col-0, *35S::ZmWRKY65* leaves under normal conditions and 24 h after incubation with *Pst* DC3000 (OD_{600}=0.002) and 10 mM $MgCl_2$.

Table S1. Cis-elements of the ZmWRKY65 promoter

Cis-element	Number
ABRE	9
MYB	19
MYC	18
LTRE	8
DRE	6

Table S2. Primers used for amplification of genes and construction of vectors

Gene/vector construction	
ZmWRKY65 -F	GAAGGCAAGAAGCAGAGGGGA
ZmWRKY65-R	CTGTGTATCTACGGGGAAAAACTGC
ZmWRKY65-RT-F	AGGGCAGTGATTGTCCAAGG
ZmWRKY65-RT-R	TGCTGAAGAGTTGCCGTCTT
ZmWRKY65-GFP-F	TATCTCTAGAGGATCCATGTCCGCGCGTCCG
ZmWRKY65-GFP-R	TGCTCACCATGGATCCATGGTATTGCTCGCT
ZmWRKY65-1302-F	GGGACTCTTGACCATGATGTCCGCGCGTCCG
ZmWRKY65-1302-R	TCAGATCTACCCATGGATGTCCGCGCGTCCG
Zm-Actin-F	GCATCCATGAGACCACCTACAAC
Zm-Actin-R	GATGGACCCTCCTATCCAGACAC

Table S3. Primers used for examination of downstream gene expression by quantitative PCR

Gene	Primers
RD29AF	GGATCAAACAGAGGAACCAC
RD29AR	GCTCCTTCTGCACCGGAAC
PR1 F	GAGGAGCGGTAGGCGTAGGT
PR1 R	CCGCTACCCCAGGCTAAGTT
PR2 F	AAGTCCATCGGACGTTGTGG
PR2 R	ACTGGGAACGTCGAGGATGA
PR5 F	TCGGGAGATTGCAAATACGC
PR5 R	ACTCTTGCAGGCCACGACAT
ERD10-F	GCAGAAGAGTACAAGAACACCGT
ERD10-R	CTCCAGTGGTCTTGGCGTGATAAC
STZ-F	CTAGTAGCGTGTCCAACTCCG
STZ-F	TTTGACCGGAAAGTCAAACCG
ACT2-F	GTGCCAATCTACGAGGGTTTC
ACT2-R	CAATGGGACTAAAACGAAAA
